# Minding the Gap: Exploring Neuroinflammatory and Microglial Sex Differences in Alzheimer’s Disease

**DOI:** 10.3390/ijms242417377

**Published:** 2023-12-12

**Authors:** Erin G. Reed, Phaedra R. Keller-Norrell

**Affiliations:** 1 Department of Pharmaceutical Sciences, Northeast Ohio Medical University, Rootstown, OH 44242, USA; 2 School of Biomedical Sciences, Kent State University, Kent, OH 44140, USA; pnorrell@kent.edu

**Keywords:** Alzheimer’s disease, microglia, neurodegeneration, neuroinflammation, sex differences

## Abstract

Research into Alzheimer’s Disease (AD) describes a link between AD and the resident immune cells of the brain, the microglia. Further, this suspected link is thought to have underlying sex effects, although the mechanisms of these effects are only just beginning to be understood. Many of these insights are the result of policies put in place by funding agencies such as the National Institutes of Health (NIH) to consider sex as a biological variable (SABV) and the move towards precision medicine due to continued lackluster therapeutic options. The purpose of this review is to provide an updated assessment of the current research that summarizes sex differences and the research pertaining to microglia and their varied responses in AD.

## 1. Introduction

Alzheimer’s disease (AD) affects approximately 6.7 million Americans aged 65 or older, of whom approximately two-thirds are women [1]. The higher number of female AD cases (the prevalence of AD) is comprised of both the number of newly diagnosed cases (the incidence of AD) and the number of individuals living with a diagnosis. Whether the incidence of AD in the US is sexually dimorphic is unclear, but women do live longer following an AD diagnosis [2,3]. This suggests not only the existence of factors that increase the overall risk in females and/or decrease the risk in males but also an increased resilience to disease processes among women diagnosed with AD. Furthermore, sexual dimorphism is seen in clinical manifestations of the disease, disease progression, neuroimaging, and pathology [4,5,6,7,8,9,10,11,12,13]. The mechanisms underlying these disparities are unknown, but research suggests a variety of candidate mechanisms, including risks linked to sex, age, gender, genetics, and environment.

**Figure 1:**
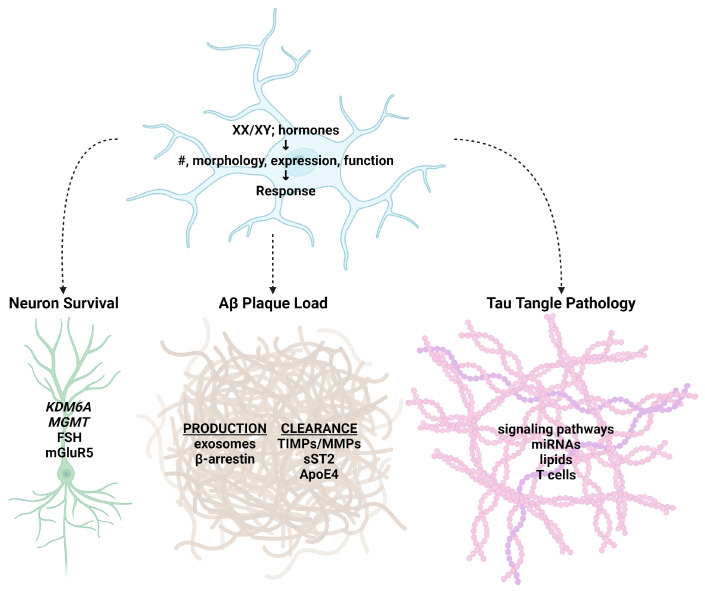
Summary figure of microglial sex differences contributing to the pathological features of Alzheimer’s disease discussed in this review. The composition of sex chromosomes (XX/XY) and/or hormones contribute to sex-specific features of microglia, including their number (#), morphology, gene and protein expression, and function. These differences may promote or inhibit sex-specific processes and pathways in neurons, amyloid plaque load, and tau tangle pathology to bias disease onset and progression in a sex-specific manner. Created with BioRender.com (accessed on 4 December 2023).

Several excellent reviews have recently been written describing microglial sex differences in brain development, maintenance, disease, and in response to lifestyle influences [14,15,16,17,18,19,20,21]. Although microglia recruit and respond to the adaptive immune system, and this interaction is becoming progressively more appreciated, the adaptive immune system in AD has been recently reviewed elsewhere [22]. We will therefore focus this review on the most recent findings, situating and contextualizing them in a rapidly expanding field to provide a comprehensive view of sex differences in AD, highlighting the contribution of microglia to these differences (summarized in Figure 1).

The authors would like to note the term sex used within this article will follow the Merriam-Webster usage guidelines (available at https://www.merriam-webster.com/dictionary/sex, accessed 29 June 2023). The authors recognize sex and gender have often been used interchangeably in western cultures, and that many languages and cultures make no distinction between the two terms [23]. However, within the context of this review, sex refers to physical traits. Gender, referring to cultural and/or societal constructs, is beyond the scope of this review. As the field continues to expand at a prodigious rate, it must coalesce around nomenclature and approaches to assess these differences to appropriately interpret and extend findings [24].

## 2. Sex Differences in AD

### 2.1. The First Patient Was a Woman

AD was first characterized by its namesake German psychiatrist, Dr. Alois Alzheimer, in 1901 when Alzheimer was introduced to Auguste Deter, a 51-year-old woman struggling with cognitive and behavioral abnormalities [25]. Alzheimer’s observations of Auguste and the subsequent evaluation of her post-mortem cerebral tissue samples by Alzheimer and two physicians, Gaetano Perusini and Francesco Bonfiglio, became the first clinical record of AD [25]. The gross histopathological observations made by Alzheimer and his team, namely the presence of neuritic β-amyloid (Aβ) plaques and neurofibrillary tangles, remain the hallmark diagnostic criteria for the final stage of AD progression [25,26].

Dr. Alzheimer’s seminal studies laid the foundation for the characterization of AD pathology in human subjects, and, in the century following his initial investigations, the origins of AD’s characteristic plaques and tangles are now understood. Additionally, new mechanisms of AD pathophysiology implicate peripheral body systems in the disorder, indicating that plaques and tangles are only one piece of the much larger puzzle that is AD.

### 2.2. Consistency across AD Subtypes

AD is categorized based on age at symptom onset and heritability. The arbitrary age of 65 years is used to distinguish early- and late-onset AD. Two or more family members having AD results in it being considered familial [27]. These categories give rise to four subtypes: familial early-onset AD (FEOAD), sporadic early-onset AD (SEOAD), familial late-onset AD (FLOAD), and sporadic late-onset AD (SLOAD). Clinical features vary between subgroups (reviewed in [28]) but overlap so substantially that subtypes cannot be clinically distinguished from each other [27]. Furthermore, common biological pathways, shared clinical and pathological features, and consistent sex effects suggest findings in one subtype (summarized in Table 1) will be relevant to the others.

In EOAD (early-onset AD), symptoms manifest prior to the age of 65 but in rare instances may begin as early as a patient’s 30s. EOAD accounts for approximately 10% of all AD patients [40]. The heritability of EOAD is 92–100% [41], and 10–15% of these familial cases are due to autosomal dominant transmission [42,43]. Numerous pathogenic loci within three genes—*Amyloid Precursor Protein* (*APP*), *Presenilin 1* (*PSEN1*), and *Presenilin 2* (*PSEN2*)—have been identified, each contributing to disease pathogenesis in a unique manner. It is important to note that mutations in these three genes only explain 5–10% of EOAD diagnoses [41,44], leaving 90–95% of EOAD cases without a definitive cause.

Among EOAD patients, females have elevated tau burden compared to their male counterparts [29]. They also present with broader atrophy associated with worse cognition at diagnosis [30]. Intriguingly, female EOAD carriers may have greater cognitive resilience to AD pathology and neurodegeneration [33] but exhibit a greater rate of neurodegeneration and memory impairment during disease progression [31,32]. The mechanisms underlying accelerated neurodegeneration in females is unknown but could be due to the sex-selective vulnerability of specific brain regions that have reciprocal connections to other regions [45,46], the interactions between tau pathology and sex-specific genes, chromosomes, and/or hormones [47], possible genetic modifying factors [28], and the sex-specific amyloid and/or tau dynamics.

Autosomal dominant mutations in *APP*, *PSEN1*, and *PSEN2* have been used to generate a variety of mouse models to recapitulate the amyloid pathology with subsequent neuroinflammation and neuron loss (reviewed in [48,49]). As in human EOAD patients, mouse models exhibit sex differences in plaque burden and rates of deposition, neuroinflammation, and cognition. While these models have extensive benefits to understanding amyloidogenic processes, they do have drawbacks including: (1) the neuronal loss in these mice is not as great as in human patients; (2) the sex effects are at times the opposite as those in human patients; (3) the absence of tau pathology, leading investigators to introduce mutations found in other tauopathies such as frontotemporal dementia to replicate that pathology. It had been proposed that the accelerated amyloid deposition in female mice was an artifact of the model, arising from the estrogen response element within the promoter used to express these mutations [50,51]. However, recent studies, some of which will be discussed in detail below, have indicated non-estrogen sensitive (i.e., sex chromosome) mechanisms may be behind some of these differences.

In contrast to EOAD, LOAD exhibits symptom onset after the age of 65 and accounts for almost all AD cases. LOAD is a complex disorder with a heterogeneous etiology and 70–80% heritability [41,52], where 40% of total LOAD cases are familial [53]. LOAD is driven by several factors including genetics, lifestyle, environment, and neurodevelopmental disorders. More than 50 genetic risk loci have been identified [54], with *Apolipoprotein E* (*APOE*) having the strongest correlation [55] followed by *Triggering receptor expressed on myeloid cells 2* (*TREM2*; [56,57]). These risk genes are associated with various biological processes, including the immune response/inflammation, lipid metabolism, and neuronal/synaptic function. Sex differences are observed in LOAD patients, with females showing faster hippocampal volume loss [12] and greater brain glucose hypometabolism [34]. Significantly, as in EOAD, females with LOAD may have greater cognitive resilience to early pathology and neurodegeneration but exhibit faster decline and progression to dementia as the disease progresses [4,6,7,12,35,36,37,38,39,58]. Several factors have been proposed as contributions to the sex differences in LOAD, including specific genes [59,60,61], inflammation [62], cardiovascular disease [63], and hormonal changes [64], although the exact mechanisms remain to be determined.

Numerous mouse models of LOAD exist. Since none of the LOAD risk alleles are independently necessary or sufficient to drive AD, investigators are generating mouse models of combinatorial risk variants [54]. As these models are developed and further characterized, it will be important to determine whether they recapitulate the sex-specific effects seen in human patients. Additional approaches involve metabolic dysregulation, traumatic brain injury, adeno-associated virus 1 (AAV1) gene transduction, toxin exposure, perturbed metal ion homeostasis, and aging (reviewed in [65]). Like in the EOAD models, many of these LOAD models show sex differences, but not all recapitulate those seen in human patients. The senescence-accelerated mouse prone 8 (SAMP8) model is of particular interest and relevance. Age is the single greatest risk factor for LOAD, and although the exact cause of accelerated senescence has yet to be determined, the SAMP8 model may shed light on the relationship between aging and AD [66], particularly in sex-specific gene regulation, as discussed below.

Sex differences occur across multiple domains in both human AD patients and in mouse models of the disease, regardless of the pathogenic mechanism. A disadvantage is that the mouse models are often only replicating a portion of the disease process, such as dominant mutations driving amyloid production. Despite these drawbacks, the consistent commonalities suggest that findings in one context may translate to others. Where there are differences between the sexes in the mechanisms underlying each type of AD, this finding provides critical avenues to develop additional therapeutics to target specific patient populations. In short, while each mouse model is an imperfect representation of the human AD condition, in a disease with such varied pathogenesis there is still much work to be done with the tools available to further our understanding and the development of effective therapeutics.

### 2.3. Accompanying Gene Expression

Not surprisingly, in both humans and mice the sex differences in disease manifestation and progression described above are accompanied by sex-specific gene expression patterns [67,68] and epigenetic profiles [69,70]. Integrative network analysis of human samples revealed sex-specific functional modules, pathways, and genes that were associated with clinical characteristics in males only and molecular networks that were more conserved temporally and spatially in females [71]. Brain-region-specific, sex-biased patterns may arise from differences in vulnerabilities and/or resilience in different brain regions at distinct stages of AD development [72]. Updates on the possible mechanisms underlying these vulnerabilities and/or resilience will be addressed in more detail in the following sections.

A gene commonly implicated across all AD subtypes is *APOE*. The predominant function of APOE is that of trafficking lipids in the central nervous system; however, it functions in several brain processes, including neuron development and function, formation of cytosolic lipid droplets, endolysosomal trafficking, mitochondrial metabolism, and innate immunity [73,74]. These varied roles contribute to the widespread effects of APOE on AD risk. There are three common isoforms—APOE2, APOE3, and APOE4—that are generated by single nucleotide polymorphisms (SNPs), resulting in differences of two amino acid residues [73]. Notably, APOE2 seems to have a protective effect, while APOE4 confers a significant risk for developing AD, although recent studies have suggested that the risks associated with APOE4 may be overestimated (reviewed in [75]). Furthermore, the biological pathways underlying the APOE4-associated risk are distinct from the protective effects of APOE2 and intersect with age-related changes in sex biology [76].

Work in mice demonstrated that ApoE is abundantly expressed in both the brain and in the periphery as distinct pools that are kept separate by the blood brain barrier (BBB). Within the brain, ApoE is predominantly produced by astrocytes, but can come from stimulated microglia [77,78,79]. In fact, in mice, microglial-derived ApoE4 signals through the ITGB8-TGFβ (integrin subunit beta 8-transforming growth factor-β) pathway to negatively regulate the microglial response to AD pathology [80]. ApoE4 produced by the liver, while not entering the brain, can exacerbate amyloid pathology [81], providing a potential peripheral therapeutic target to treat AD.

In human AD patients and in mouse models, females are more strongly affected by APOE status. Female-specific effects due to APOE4 are seen in cognitive impairment [82], tau accumulation [83], gene expression [84,85], brain metabolism [86], cerebral microbleed frequency [87], TREM2-dependent microglial activation [88], and astrogliosis [89]. This could be due in part to the APOE4 modulating expression of the estrogen receptor ERα. It has been proposed that males and females share genetic regulators of amyloidosis, but, as amyloid pathology progresses, it has been demonstrated that APOE exerts sex-specific effects on gene expression, resulting in sex bias in disease manifestation [60,61].

A top key regulator of female AD gene networks was determined to be lipoprotein receptor-related protein 10 (LRP10), potentially driving sex differences based on its high regulatory strength and network connectivity, sex-specific differential expression, and dependence on APOE4 gene dosage [72]. It is hypothesized that reduced LRP10 in APOE4-carrier female AD brains increases the amyloid burden through enhanced amyloid production and reduced clearance due to impaired protein trafficking [72].

Epigenetic modifications including methylation and acetylation regulate chromatin structure and gene expression without modifying the DNA sequence. They are important contributors to sex differences in brain function and AD vulnerability [90,91,92,93,94,95,96,97,98,99,100]. Significantly, they are modifiable, transmissible, and strongly influenced by environmental factors including hyperphosphorylated tau [101,102,103,104,105]. Epigenetic changes, including chromatin-modifying enzymes, epigenetic marks, and microRNAs (miRNAs), are observed in humans during normal aging and AD, and are found in the SAMP8 mouse model, potentially accounting for the accelerated senescence (reviewed in [106]). Sex-specific epigenetic changes in the dorsal hippocampus, a region important for learning and memory, suggest epigenetic mechanisms in this region are regulated in sexually dimorphic ways [70]. Zhang et al. recently conducted a comprehensive meta-analysis revealing several novel DNA methylations associated with the AD Braak stage in a sex-specific manner [69]. In fact, for many genes previously implicated in AD neuropathology, the effects are predominantly driven by only one sex, with enrichment of integrin activation in females and complement activation in males [69].

These studies provide important insights into the molecular contributions underlying sex differences in AD onset and disease progression. However, while many studies did evaluate regional differences, cell-type-specific changes were largely absent. This is significant as each brain cell type contributes to brain function in disease in a unique manner. Furthermore, various cell types may work in concert or in opposition to other cell types in response to pathology.

## 3. Sex-Specific Neuroinflammation

Inflammation and neurodegeneration are strongly correlated, which is evidenced by the presence of inflammatory cytokines in the brains and CSF (cerebrospinal fluid) of AD patients. These are produced in response to several AD-related processes, including amyloid plaques, neurofibrillary tangles, and signals coming from sick, dying, and dead neurons [107]. As these stimuli are never completely removed, the inflammatory response becomes chronic, exacerbating the pathology and furthering disease processes. The significance of this neuroinflammatory response is highlighted by the substantial number of immune-related genes found to be correlated with AD risk in several genetic studies.

### 3.1. Sexually Dimorphic Microglia

The cells primarily responsible for this inflammatory response in the AD brain are microglia. They derive from peripheral macrophages that infiltrate the brain early in embryonic development prior to the formation of the blood–brain barrier [108,109]. These microglial progenitors proliferate and colonize the brain, tiling themselves such that no two microglia survey the same brain area. This colony of cells continues to proliferate until the cells reach optimum density [108,110]. Throughout development and adulthood, microglia serve a variety of homeostatic needs, ranging from synaptic pruning to acting as macrophages when cells undergo apoptosis [111,112,113]. They secrete both pro- and anti-inflammatory cytokines in a context-dependent manner, as well as neurotrophic factors [112].

Microglia actively survey the brain parenchyma, constantly extending and retracting processes such that the entire brain parenchyma is assessed within a few hours [114]. Following disruption by injury, infection, or disease, microglia shift from this active surveillance state to one that is responsive to the disruption. This shift is reflected by a general change in their morphology, and the acquisition of distinct transcriptional profiles. With the advent of advanced techniques, a more comprehensive understanding of these phenotypes has led to a recent re-evaluation of microglial nomenclature and classification [115].

Whether microglia exhibit sex differences under homeostatic conditions has been under active investigation and has yielded sex-specific patterns that depend on brain region and age (summarized in Table 2). There are no differences in microglial number or density in the amygdala, hippocampus, or prefrontal cortex during the embryonic or early-neonatal periods [16,116,117]. During the first postnatal week, males have more phagocytic amoeboid microglia in the amygdala, while females have more in the hippocampus [16,116,118]. Microglia of the dentate gyrus do not exhibit sex differences in their number, density, or morphology at post-natal day 10 (P10) [119]. However, by adolescence, morphological sex differences are accompanied by sex differences in microglial density. Male microglia in the prefrontal cortex have more complex branching, particularly in the branches more proximal to the soma [117], and a higher density in the cortex and hippocampus, compared to a higher density in the amygdala in females [120]. Microglia in the hippocampus of females are larger and more phagocytic than those in males [121]. Between adolescence and adulthood, another shift occurs, where the branching of female microglia in the prefrontal cortex becomes more complex [117]. Males continue to have a higher density of microglia in the hippocampus and cortex [120]. However, using hierarchical clustering on principal components, a recent study of the microglia of the adult mouse central nervous system found no sex differences in microglial morphology [122]. These discrepancies in microglial density and morphology could arise from a variety of sources, including pathogen exposure in housing, tissue processing and immunostaining techniques, and quantitative approaches. Moreover, sex and strain differences have been noted in other immune cell types such as B and T lymphocytes, NK cells, immature myeloid cells, and macrophages [123], suggesting that evaluation of various mouse strains may further complicate both comparison and interpretations.

While the general features of microglia (i.e., their number and shape) provide conflicting evidence of sex differences, expression studies are much more definitive, indicative of latent sex differences where male and female microglia use different mechanisms (such as gene expression and signaling cascades) to achieve similar outcomes (including the number, shape, and response to insult). At embryonic day 14.5 (E14.5), microglia from male and female mice are not transcriptionally different from each other [124]; however, distinct transcriptomes are seen at E18.5, with enrichment of apoptotic and inflammatory genes in female microglia [125]. Homeostatic microglia from male and female neonatal and adult mice continue to express different genes [120,126,127,128,129] and miRNAs [130], such that the inflammatory milieu of the brain is different [131]. Notably, marginal sex differences were observed in the gene expression profiles of hippocampal microglia from young mice, but sex chromosomally and autosomally encoded differences emerged with aging, with a female bias towards senescence and inflammation [132]. These studies have led to the hypothesis that male and female microglia mature at different rates, with implications for their response to various stimuli. In fact, after immune challenge, there are sex differences in the cytokines found in the hippocampus [133] and in discrimination memory impairments [134]. Microglia migrate at sex-specific rates [120], and, after injury, microglial mobility is regulated by interferon (IFN) γ in males but not females [135]. Single-cell and bulk RNA sequencing have also provided evidence for sex-specific gene expression in human microglia [136], extending the relevance of the findings in mice to humans.

**Table 2:** Sex differences observed in microglia across the rodent lifespan.

Age *	Species	Microglial Sex Difference	References
E14.5	Mouse	No difference in transcriptome	[124]
E17	Rat	No difference in number, morphology in amygdala, hippocampus	[116]
E18.5	Mouse	Females: express more apoptotic, inflammatory genes	[125]
Birth/P0	Rat	No difference in morphology in prefrontal cortex	[117]
P0–P4	Rat	Males: higher density in amygdala	[16]
P3	Rat	Females: more phagocytic in hippocampus	[118]
	Mouse	Females: express more inflammatory cytokines	[126]
P4	Rat	Males: more amoeboid in cortex, hippocampus, amygdala	[116]
P8	Mouse	Females: larger, more phagocytic in hippocampus	[121]
P10	Mouse	No differences in number, density, morphology in dentate gyrus	[119]
3 weeks	Mouse	Males: higher density in hippocampusFemales: higher density in amygdalaNo difference in density in striatum, cerebellum	[120]
P28	Mouse	Males: larger, more phagocytic in hippocampus	[121]
P30	Rat	Females: more activated in cortex, hippocampus, amygdala	[116]
		Males: more complex branching in prefrontal cortex	[117]
P60	Rat	Females: more activated in cortex, hippocampus, amygdala	[116]
	Mouse	Females: more transcriptionally mature	[127]
	Mouse	Females: increased inflammatory gene expression	[127]
2–6 months	Mouse	Males: IFN-dependent migration after injury	[135]
12 weeks	Mouse	Males: express more inflammatory genesFemales: more neuroprotective	[129]
P90	Rat	Females: more complex branching in prefrontal cortex	[117]
3 months	Mouse	No difference in morphology in any brain, spinal cord region	[122]
13 weeks	Mouse	Males: higher density in hippocampus, cortex, amygdalaNo difference in density in striatum, cerebellumMales: greater antigen presentation capability	[120]
18 months	Mouse	Females: more phagocytic, reduced ability to respond to insult	[137]
22–25 months	Mouse	Females: express more disease, senescence genes	[132]
24 months	Mouse	Females: express more inflammatory genes	[128]

* E, embryonic day; and P, postnatal day.

### 3.2. Sources of Microglial Sex Differences

The mechanisms giving rise to these sex differences in microglial gene expression and responses are currently being investigated and include both genetic and hormonal influences. Significantly, sex chromosomes and sex hormones can act in a synergistic or antagonistic manner on a given process [138].

From a genetic standpoint, every cell in the body, microglia included, contains a set of sex chromosomes. Placental mammalian females have two X chromosomes, and placental mammalian males have an X and a Y chromosome. As the X chromosome contains a plethora of genes, many with immune-related functions, and a high concentration of microRNAs that can regulate expression of autosomal genes, the expression of XX chromosome gene dosage must be normalized to the XY levels in males. This is accomplished through the action of the long non-coding (lnc) RNA *Xist* in a process referred to as X chromosome inactivation (XCI). Detailed reviews of this complex process have been recently published [139,140,141,142], so we will only summarize it here.

*Xist* is randomly transcribed from one X chromosome; one to two RNA molecules recruit chromatin-modifying proteins, transcriptional silencers, and other RNA-binding proteins to approximately 50 distinct foci along one X chromosome; subsequently, local protein gradients are generated to coat and render that X chromosome inactive (Xi; [139,140,141,142,143]). Interestingly, San Roman et al. found that the active X (Xa) and inactive X (Xi) transcriptomes to be modular, with Xi modulating Xa transcript levels in *cis* and in *trans* [144]. Furthermore, only ten X chromosome genes were identified as most likely to contribute to male–female differences in common diseases [144], providing critical insight into the expression of genes on the X chromosomes. The escape or disruption of XCI leads to the loss of gene dosage compensation, driving pathogenic immune responses [145]. X chromosome epigenetics may relate to an elevated AD risk [146], where there is a robust neuroinflammatory response contributing to disease onset and progression. Furthermore, the extent of XCI, particularly in the brain, decreases with age [147], further increasing X chromosome-related gene expression.

Another important event during aging that can alter expression from the sex chromosomes is loss of the Y chromosome (LOY). An increasing frequency of mitotic missegregation errors along with declining genomic instability and impaired DNA repair capabilities may lead to LOY [148,149,150]. This common post-zygotic structural mutation shows a robust association with AD [151,152], contributing to disease through immune system dysfunction [150,153,154]. It has been hypothesized that as microglia proliferate, they could be more prone to LOY accumulation [155]. A recent study showed LOY is enriched in microglia from AD patients, resulting in dysregulation of many genes associated with aging and inflammation [156]. This study demonstrates how perturbations in processes associated with age-related inflammation could lead to neurodegeneration.

Hormones, including gonadotropins, androgens, and estrogens, also contribute to brain sexualization (reviewed in [18]). During critical windows in the neonatal period, sequential surges of these hormones result in the life-long patterning of brain circuitry that can then be re-activated by circulating hormones in adulthood. Microglia are affected by these neonatal surges, as administration of estradiol or testosterone to female rodents during these critical windows phenotypically and molecularly masculinizes the microglia, as well as masculinizes rodent behavior in adolescence and adulthood ([117,129,157]). Furthermore, administration of indomethacin to males prevents the masculinizing effects of prostaglandin E2 [158,159,160]. Epigenetic mechanisms prevent masculinization of the brain; however, the details of how this occurs remain elusive [21]. Interestingly, a recent study suggested estrogens may restrain microglial immune responses, thereby reducing vulnerability to adverse behavioral changes [161].

### 3.3. Microglial Responses in AD

Upon recognition of injury or insult, microglia transition to a responsive state with sex and age playing a role in this response [128,129,162]. Single-cell and single-nuclei sequencing studies have rapidly expanded the understanding of diverse microglial responses to AD-related pathology in human patients and in rodent disease models. Notably, female mice indicate sex-dependent microglial activation in response to amyloid but not tau pathology [163]. Although some differentially expressed genes in microglia are common between amyloid and tau pathologies, microglia upregulate phagocytic, inflammatory, and proteostatic pathways in areas of higher amyloid and interleukin (IL)-1 in association with tau [164]. The gene signature of disease-associated microglia (DAM), also referred to as neurodegenerative microglia (MGnD), is strongly correlated with apoptosis and myelin debris, consequences of neurodegeneration and inflammation, and is associated with amyloid pathogenesis [165,166,167,168]. Initially, DAMs/MGnDs associate with amyloid plaques, aiding in amyloid clearance though inefficiently [165,169]. Their prolonged presence contributes to the pathogenesis of neurodegenerative diseases through the impairment of microglial homeostatic mechanisms such as phagocytosis, antigen presentation, cell motility, dysregulation of reactive oxygen species (ROS) generation, and increased cytokine expression, resulting in neuron loss [165,166]. Notably, sex differences exist within these gene signatures (reviewed in [170]), but how they arise and whether they induce or are secondary to disease processes is not entirely clear and is being assessed.

It is becoming increasingly appreciated that early-life adverse events or conditions may affect microglia such that they later behave in ways that promote AD pathogenesis. A common approach to studying these effects is through neonatal maternal separation (MS) of rodent pups from their mother. This early-life adversity drives sex-specific changes in microglial morphology and immune challenge responses at various ages [171]. MS also alters cortical microglial activation, hippocampal gene expression, synaptic markers, and immune cell populations in sex-specific ways at various ages, and exacerbates amyloid deposition, particularly in females [172,173]. Additionally, neonatal immune challenges result in female-specific changes in social behavior and microglial cell number [174]. A recent study reported that even prenatal environmental stressors activated the immune system such that postnatal microglial function and adult behavior was impaired in males [175]. Together, these findings indicate that developmental and/or early-life events can program microglia to respond in distinct ways, with implications for subsequent responses during adulthood.

## 4. Sex-Specific Impacts on Brain Cytoarchitecture

As mentioned above and recently reviewed extensively elsewhere, the brain is highly sexualized across multiple levels, from genes to cells, circuits, and behaviors [176,177,178]. Both sex chromosomes and signaling through gonadal hormone receptors affect brain structures and gene regulation in humans and mice [170,179,180]. Recent studies have reported sex-specific microglial regulation of cell genesis in the neonatal hippocampus [181], sexual dimorphism in stereotyped cell-type-based cortical architecture [182], hippocampal astrocytes [183], and oligodendrocyte precursor cells [184]. Sex differences exist in brain protein expression [185] and gene expression and behavior [186]. Furthermore sex-specific reliance on certain proteins impacts synaptic connectivity, microglial activity, and behavior [187].

### 4.1. Sex-Specific Neuronal Effects in AD

Female AD patients have greater brain atrophy and neurodegeneration than male AD patients, contributing to greater declines in memory, reasoning, language, and spatial orientation [2,7,10,35,188,189,190,191]. As discussed extensively elsewhere [192,193,194,195], olfactory dysfunction has been reported in human AD patients and in mouse models of AD. It is one of the earliest clinical symptoms of AD, thereby acting as a biomarker of disease, and exhibits a strong sex bias. Changes in brain region volumes and connectivity as well as neurotransmitters have been implicated in olfactory deficits (reviewed in [196]). In fact, women exhibit accelerated age-related loss of olfactory cortical neurons [197]. Gene expression (sex chromosomal and autosomal) and hormones have recently been identified as potential mechanisms that could contribute to sex differences in neuronal survival during AD pathogenesis.

As discussed above, in females one X chromosome must be silenced or inactivated to achieve proper gene dosage. However, some genes on the Xi escape this silencing and may contribute to or modulate neurodegenerative processes. One such gene is *KDM6A*, also known as *Utx*, which encodes a lysine-specific histone demethylase important in cognition [198,199,200,201,202,203,204,205,206]. XCI escape by *KDM6A* increases its expression in the brains of females, conferring resilience to AD-related neuronal vulnerability [3]. This effect is independent of its demethylase function and could provide novel therapeutic targets for treating cognitive deficits in both sexes [207].

Another gene important in neuronal survival is *MGMT*, which encodes a DNA methyltransferase that is important in protecting cells from apoptosis following DNA damage [208]. Women, particularly those who do not carry the APOE4 allele, express less MGMT and are more likely to have AD [209]. The lower expression of MGMT stems from sex-specific methylation patterns due to SNPs in the MGMT locus that interact with distal enhancers via chromatin loops [210,211]. The mechanisms underlying sex-specific methylation patterns and SNPs remain to be determined. The interaction of MGMT with APOE may occur due to convergence on signaling pathways associated with inflammation.

The neuroprotective effects of estrogen are widely appreciated, but recently the pituitary gonadotropin follicle-stimulating hormone (FSH) was implicated in female susceptibility to AD [212]. FSH levels rapidly increase in the perimenopausal phase, and elevated levels are strongly associated with the onset of AD [213,214,215]. FSH-induced neuronal apoptosis in a mouse model of AD and blocking FSH activity using an anti-FSH antibody prevented neuron loss [212]. These effects on neuronal survival were accompanied by changes in synapse number, cognition, and amyloid and tau pathology, revealing a new potential mechanism underlying the accelerated AD pathogenesis in women during menopause, providing another potential therapeutic target.

In addition to neuronal survival, neuronal activity also exhibits sex specificity. Glutamate is the main excitatory brain neurotransmitter and plays a key role in learning in memory [216,217]. In males but not females, the G-coupled metabotropic glutamate receptor mGluR5 on neurons tightly binds oligomeric amyloid in a cellular prion protein-dependent manner, suppressing autophagic signaling, resulting in cognitive deficits [218]. These sex-specific effects in mGluR5 binding and scaffolding are estrogen-independent, but the source of this difference remains to be determined. This study indicated the possibility of repurposing mGluR5-selective modulators to treat male AD patients, highlighting the need to stratify clinical trials assessing AD therapeutics by sex.

### 4.2. Sex-Specific Glial Effects in AD

Glia modulate neuronal activity. Astrocytes are the most abundant glial cells, playing critical roles in synaptic transmission and plasticity by providing trophic and metabolic support to neurons. Female hippocampal astrocytes in a mouse model of AD exhibited low inflammatory activity and calcium flow associated with low cannabinoid signaling compared to their male counterparts [219]. These sex differences were evident at birth, suggesting intrinsic sex differences in astrocyte activity that may eventually impact their response during disease progression.

Microglia also modulate the brain’s activity through removal of synapses as well as entire neurons. A subset of microglia, ARG1+ microglia, located primarily in the basal forebrain and ventral striatum during early-postnatal development in the mouse, were recently reported [220]. These microglia are enriched in phagocytic inclusions, exhibit a distinct molecular signature, and play a critical role in shaping neuronal circuits involved in cognition through their actions on cholinergic innervation and spine maturation. Microglia also communicate with astrocytes providing neuronal support and synapse pruning, as discussed above. How microglial functions in homeostatic and AD processes interact with recently identified chromosomal and hormonal factors contributing to neuronal resilience and protection remains to be determined.

**Figure 2:**
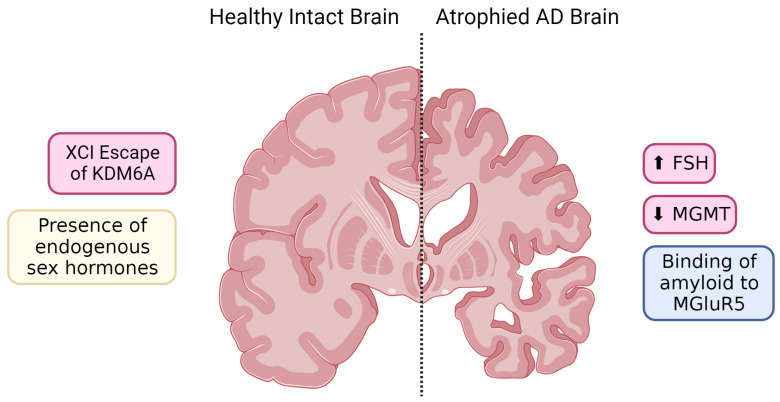
Sex-specific expression patterns of transcription factors, follicle-stimulating hormone (FSH), and mGluR5 contribute to differential patterns in neuronal survival and overall brain atrophy. Pink boxes represent female-specific effects, while blue boxes represent male-specific effects. Arrows represent altered expression levels contributing to AD risk/presentation. Yellow boxes represent processes that exist within both sexes. Created with BioRender.com (accessed on 4 December 2023).

Estrogens and androgens have long been understood to have neuroprotective and anti-inflammatory effects. However, together, these recent findings illustrated in Figure 2 provide novel mechanisms contributing to neuronal resilience and vulnerability, whether it be intrinsic to the neurons themselves, or secondary to glial-mediated effects. Autosomal or sex chromosomal genes are now appreciated to have effects on neurons and glia, and the identification of another hormone provides another potential therapeutic target.

## 5. Sex Differences in Amyloid Deposition

Aβ was first implicated in the pathogenesis of neurocognitive disorders in 1984, when meningovascular plaques from the brains of Down’s Syndrome patients were genetically sequenced and found to be comprised primarily of Aβ [221,222]. Subsequent studies sequenced the *APP* gene and confirmed that Aβ originated from the biochemical processing of APP. By 1991, a large body of evidence suggested that the plaques observed in AD were like those initially observed in Down’s Syndrome patients [222,223,224]. Females exhibit a greater amyloid burden compared to males at comparable disease stages in both humans and mice, which could arise from differential rates of amyloid production and/or clearance.

### 5.1. Aβ Production

Aβ is formed through sequential proteolytic processing of the transmembrane Amyloid Precursor Protein (APP), which is ubiquitous throughout the central nervous system (CNS; [224]). In healthy brains, cleavage of APP by α-secretase does not produce pathogenic Aβ but rather soluble byproducts [225]. In AD, APP is cleaved by β-secretase (BACE), resulting in two soluble ectodomains and two carboxy-terminal fragments [224]. These fragments are further cleaved by γ-secretase, leading to the production of pathogenic Aβ, p3, and the intracellular APP domain [224,226]. γ-secretase is a multi-subunit protease containing Nicastrin, Presenilin 1 and/or 2 (PSEN1/PSEN2), Presenilin 2 Enhancer (PEN2), and Anterior Pharynx-Defective 1 (APH-1). The Presenilins form the catalytic subunit of γ-secretase; thus, mutations in PSEN1/PSEN2 increase the activity of the γ-secretase proteolytic pathway, increasing the deposition of Aβ [227,228,229].

Aβ has two major isoforms found in AD, Aβ_40_ and Aβ_42_, with the latter linked to the deposition of neuritic plaques [230,231,232]. AD patients exhibit an increased Aβ_42_:Aβ_40_ ratio, which is thought to drive protein self-aggregation, contributing to Aβ plaque formation [233,234]. The accumulation of Aβ in AD follows a characteristic pattern, beginning with the neocortex [226,235]. As the disease progresses, Aβ accumulates in progressively deeper structures, ending with the brainstem [226,235]. Aβ deposition is thought to be the earliest pathology to develop in AD, preceding the onset of clinically observable symptoms by decades, though this hypothesis remains debated [236,237,238]. The Amyloid Hypothesis suggests Aβ deposition contributes to the development of later AD symptoms. However, the precise mechanisms underlying this catalysis remain to be determined, and the hypothesis has been serially revised with new findings.

Though plaque load does not correlate well with cognitive deficits, the amyloid cascade hypothesis suggests greater amyloid load eventually results in more significant disease. In humans, there is an association between antecedent attention-deficit/hyperactivity disorder and increased risk of dementia [239,240]. Within the striatum, female 5xFAD mice exhibit increased amyloid plaque associated with changes in dopamine signaling in the dorsal striatum, thereby resulting in hyperactivity of female but not male mice [241].

Some AD patients also exhibit cerebral amyloid angiopathy (CAA), which involves accumulation of amyloid protein in the leptomeningeal and cortical blood vessels. Interestingly, men have more severe CAA than women [242]. Perez et al. have recently shown that the transcription-regulating protein inhibitor of the DNA-binding protein 3 (ID3) is associated with CAA severity in women, while the nuclear respiratory factor 1 (NRF1) is associated with CAA severity in men [243], potentially providing new targets for personalized medicine and/or prevention strategies against CAA.

Secretion of Aβ via exosomes, which are small vesicles derived from the inward budding of intraluminal vesicles (ILVs) inside multiple vesicle bodies (MVBs; [244]), following its production through the series of enzymatic steps described above, promotes plaque formation in a variety of model systems (reviewed in [245]). Ceramides are integral components of cell membranes, and the sphingolipid ceramide neutral sphingomyelinase (nSMase) promotes the formation of exosomes [246,247]. In an AD mouse model, female but not male mice exhibit elevated ceramide and exosome levels and are uniquely sensitive to nSMase inhibition, which blocked exosome spreading and subsequent amyloid pathology and rescued cognition [248]. The reason that ceramide and exosome biogeneses have a more significant role in the amyloid pathology of females is unclear and remains to be determined.

Furthermore, stress modulates amyloid production and does so in sex-specific ways. Adult female APP/PS1 mice show significantly increased hippocampal Aβ, while males do not, due to differences in β-arrestin involvement in corticotropin-releasing factor receptor signaling pathways [249].

The relationship between hormones and amyloid levels is complex and seemingly contradictory. The primary estrogen, 17β-estradiol (E2), decreases Aβ production [250,251,252] and stimulates its degradation [253]. This would suggest that females, having higher concentrations of circulating E2, would have decreased amyloid burden compared to males, when in fact the opposite is true. Future work must tease apart the relative contributions of and the balance between estrogenic effects on amyloid production and clearance and its influence on other cellular processes that could in turn modulate those effects, such as neuronal survival and neuroinflammation.

### 5.2. Aβ Clearance

Early-onset forms of AD are thought to arise from the increased production of Aβ, while late-onset forms stem from reduced Aβ clearance [254,255]. This clearance occurs through both enzymatic and non-enzymatic processes (reviewed in [256]).

In the brain, Aβ is primarily degraded and cleared through the proteolytic machinery [257,258], and more than 20 different Aβ-degrading enzymes (ADEs) have been identified, including metallo-serine, aspartyl, cysteine, and threonine proteases [259]. To date, metalloproteases are the best studied ADEs, with matrix metalloprotease 2 (MMP2), MMP7, and MMP9 demonstrating Aβ-degrading activity and association with AD [256]. Tissue inhibitors of metalloproteases (TIMPs) regulate MMP activity [260] and have been linked to AD as well [137,261,262,263]. Notably, MMPs may have sex-specific mechanisms contributing to AD [264], potentially due to their interactions with estrogen [265]. Aksnes and colleagues found that amyloid pathology is associated with MMP3 in males but with TIMP4 in females [266]. Furthermore, there was a female-specific effect of MMP10 on cognitive and functional decline in AD patients, suggesting that MMPs and TIMPs could be useful biomarkers for sex differences and progression in AD [266].

Non-enzymatic clearance of Aβ occurs through a variety of pathways, including microglial phagocytosis (reviewed in [267]). As microglia age, they become senescent/dystrophic, exhibiting age-related changes in the expression of phagocytosis-related receptors and processes, limiting their capability to respond to pathogens or neurodegenerative processes [268]. Microglial aging is accompanied by increased phagocytosis of neuronal debris, with female microglia exhibiting a greater increase but a loss in ability to adapt its phagocytosis to inflammatory conditions [269]. A recent report found sST2, the secreted isoform of the interleukin-3 (IL-33) receptor, is a decoy receptor that inhibits microglial activation, increases in the blood and brain of females with AD, and is positively associated with disease progression [270]. Furthermore, women with APOE4 that have lower amounts of sST2 have a lower risk of AD [270]. The authors hypothesize that less sST2 facilitates microglial clearance of Aβ from the brain, thereby reducing AD.

Recently, two companion studies demonstrated that microglial-derived ApoE4 was detrimental to the microglial response to amyloid, and this was more pronounced in females [80,271]. ApoE is essential for seeding amyloid deposits [272,273,274] as well as being required for microglial conversion to a DAM/MgnD phenotype in the presence of amyloid [165,169]. Using different genetic and molecular approaches, these studies showed microglial ApoE3 induced microglial DAM/MGnD genes for plaque encapsulation and clearance, while microglial ApoE4 induced ITGB8-TGFβ signaling that impaired this DAM/MGnD response, thereby exacerbating amyloid pathology [80,271]. In both studies, female AD patients were more strongly affected by the presence of an APOE4 allele in these processes, though the mechanism behind this sex-specific effect was unclear.

**Figure 3:**
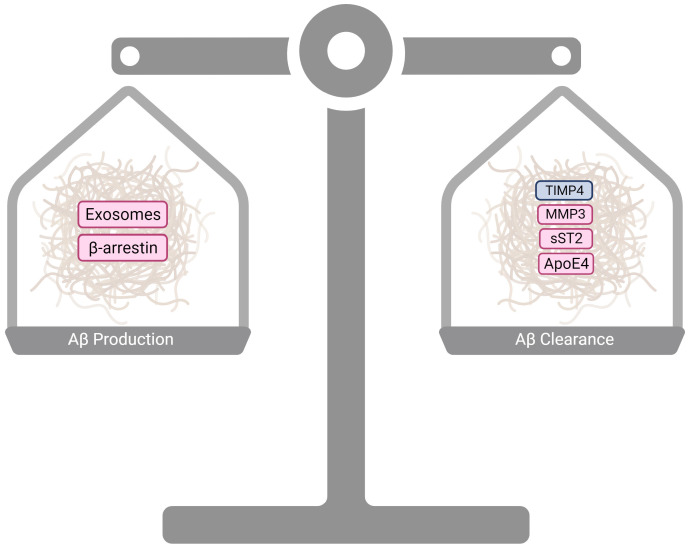
Sex-specific processes contributing to differential amyloid plaque load through either Aβ production or Aβ clearance mechanisms. Burgundy boxes represent female-specific processes, while blue boxes represent male-specific processes. Created with BioRender.com (accessed on 4 December 2023).

Together, recent findings summarized in Figure 3 suggest sex differences in microglial inflammatory response mechanisms may contribute to distinct inflammatory profiles, thereby differentially stimulating amyloid production and/or modulating its clearance. ApoE is likely to function in many of these processes, participating not only in amyloid clearance and plaque seeding directly but also in the acquisition of microglial phenotypes that facilitate or inhibit amyloid production and clearance. Future work will need to elucidate mechanisms contributing to sex differences in microglial responses generally and ApoE4 sensitivity specifically, as they could provide avenues for more targeted interventions and therapies.

## 6. Sex Differences in Tau Pathology

The third pathological hallmark of AD is intraneuronal tangles composed of the hyperphosphorylated microtubule-associated protein, tau. Unlike amyloid plaques, tau pathology is well correlated with brain atrophy and cognitive decline [275,276,277].

### 6.1. Intracellular Tau Tangle Production

*Microtubule Associated Protein Tau* (*MAPT*) encodes the cytoskeletal phosphoprotein tau, which encourages the formation of microtubules from tubulin and is normally found in mature neurons [278]. The transcription of *MAPT* can result in six different tau isoforms, with varying numbers of microtubule-binding repeats and amino-binding terminal inserts [278]. Tau shows highly variable structural confirmations, only exhibiting conformational consistency in β pleating between microtubule-binding repeats [278]. Under normal physiological conditions, the ratio of tubulin to tau in mature neurons is approximately 10:1. With tau’s high binding affinity to tubulin, little tau remains unbound in healthy adult cells [278]. As aforementioned, tau is a phosphoprotein, with over 40 different sites where phosphorylation may occur [279]. The normal concentration of bound phosphate relative to the tau protein is 2–3 mol phosphate/1 mol of protein; however, in AD patients, tau exhibits a 3–4-fold increase in bound phosphate concentration. This inhibits protein function and induces tau aggregation, leading to the formation of neurofibrillary tangles (NFTs; [278,280,281]). Although NFTs may develop in AD patients, most of the hyperphosphorylated tau (hTau) generated remains in the cytosol, where it sequesters functional tau proteins and disrupts the formation of microtubules [278]. Both NFTs and cytosolic hTau disrupt normal cell function and lead to cognitive decline; however, when hTau is dephosphorylated, it returns to a normal, functional state, supporting the creation of microtubules [278,282].

As in neuronal loss and amyloid burden, sexual dimorphism is evident in tau deposition and tau-related clinical progression, with women being more strongly affected [12,38,283,284,285,286,287,288]. Higher tau levels occur in women, particularly in the context of elevated Aβ and especially in the context of an earlier age of menopause and late initiation of hormone replacement therapy (HRT; [289]).

### 6.2. Microglial Responses to Tau

Despite tau being a structural neuronal protein and contributing to intraneuronal neurofibrillary tangles, it propagates from neuron to neuron, where it can activate microglia (reviewed in [290]). Tau activates a variety of signaling cascades in microglia, including the Toll-like Receptor 4 (TLR4)- NOD-, LRR- and pyrin domain-containing 3 (NLRP3)-caspase 1 cascade for phagocytosis of living neurons [291]. Additionally, the cyclic GMP-AMP synthase (cGAS)-IFN signaling pathway suppresses MEF2C neuronal transcriptional networks to attenuate cognitive resilience [292]. Microglial NFκB signaling also drives tau spreading and toxicity [293]. The kinase Tumor progression locus 2 (TPL2; [294]) and insulin-like growth factor-binding protein like protein 1 (IGFBPL1; [295]) function as master regulators of the microglial inflammatory responses to tauopathy. Sex-specific microglial signaling pathways and miRNAs contribute to differences in the microglial transcriptome, perturbations in lipid metabolism and lipid-droplet accumulation, and differential recruitment of T cells, all of which may contribute to sex-disparate tau pathology [130,296,297].

### 6.3. Reversal/Removal of Pathological Tau

Though tau pathology is reversible experimentally, clinical trials of therapies targeting tau pathology have not yet yielded positive results, though candidate compounds continue to make their way through the clinical trial pipeline [298,299]. Further, tau pathology is intimately linked with the presence of Aβ plaques in AD, with Aβ clearance often aiding in the clearance of hTau in the early stages of the disease [300,301]. The mechanism underlying this close pathological relationship remains poorly understood, although some hypotheses point toward the proteasome [300,301,302].

Tau clearance occurs through the ubiquitin–proteasome system and the autophagy-lysosome pathway, with the final modification being ubiquitination [303,304,305]. The ubiquitin-specific peptidase 11 (USP11) deubiquitinates tau, augmenting its aggregation [306]. USP11 is an X-linked gene that escapes complete XCI, resulting in elevated USP11 expression in females, correlating strongly with tau pathology in females but not males; therefore, it potentially underpins heightened disease susceptibility in women [306].

There has been a significant shift in focus away from amyloid to tau pathology. The focus on amyloid has been called into question due to failure of anti-amyloid therapeutics in the clinic, and the appreciation for high correlation between tau levels and cognitive decline. Recent work has illuminated the response of microglia to tau and has begun to demonstrate sex-specific microglial responses, potentially contributing to distinct disease manifestations. Many questions remain to be answered, most notably the role of APOE4. As in amyloid pathology, APOE4 from neurons and astrocytes play important roles in the tau-mediated gliosis and neurodegeneration [307,308]. What remains to be determined is whether these effects are more acute in females compared to males, as in amyloid pathology.

## 7. Conclusions and Future Directions

In the century following Dr Alzheimer’s encounter with Auguste Deter, there has been substantial progress in understanding the basic mechanisms of the three major pathological hallmarks of AD: neuron loss, extracellular amyloid plaque deposits, and intracellular tau tangles. Furthermore, inflammation mediated by the innate immune cells of the brain, the microglia, is understood to be a key influence on those hallmark pathologies. While the disparate impact of AD on women has long been widely appreciated, the reasons for sex-specific disease onset and progression remain elusive. Early work focused on the neuroprotective and anti-inflammatory aspects of hormones. More recently, genetic effects driven by sex chromosomes and epigenetics have been shown to play a role in neuronal resilience and activity, and microglial-mediated amyloid and tau clearance mechanisms. Despite these advances, much work remains to be done, both in understanding sex-specific mechanisms and in leveraging these findings for a personalized approach to preventing and treating AD.

The mechanisms behind the increased sensitivity of women to the APOE4 isoform remain a significant gap in our understanding. This has major implications on disease, as APOE has such varied functions, from lipid transportation to amyloid clearance and microglial responses to amyloid and tau. The complex interplay of APOE isoforms and sex were highlighted in a recent paper demonstrating in mice that the ApoE-isoform and microbiota-dependent progression of tau pathology occurred in a sex-specific manner [309]. Whether targeting gut microbiota may provide a potential preventative or therapeutic approach to AD in one or both sexes remains to be determined.

Metabolism is another avenue ripe for future investigation. The AD brain exhibits metabolic dysregulation, leading to the Metabolic Reprogramming Theory of AD [310]. In human patients and in mouse models of the disease, there are sex differences in cerebrovascular and brain metabolism [311,312,313]. In fact, elevated brain metabolism in women is hypothesized to confer cognitive resilience against early-onset AD [36]. Furthermore, dietary fat is known to influence microglia [314], and a high-fat, high-salt diet induces sex-specific responses in the gut microbiome and in hypothalamic astrocytes and microglia [315]. Daily administration of an insulin-sensitizing compound ameliorated female-specific metabolic imbalances in a mouse model of AD [316]. Whether using drugs targeting genes associated with both lipid metabolism and neuroinflammation [317] will be efficacious in one or both sexes, if at all, remains to be determined.

Numerous other avenues for additional work exist and understanding them is required. AD, particularly late-onset sporadic forms, is incredibly heterogeneous and will require multiple approaches to effectively treat the increasing patient population. Some therapeutic targets will be consistent across groups of patients, such as ApoE, but how they are targeted and how effective they may be is likely to depend on sex and a variety of other intersecting factors.

## Figures and Tables

**Table 1:** Sex differences in clinical phenotypes observed in AD subtypes.

AD Subtype *	Clinical Metric	Sex Difference *	References
EOAD	Tau burden	F > M	[29]
	Brain atrophy, cognition at diagnosis	F > M	[30]
	Rate of neurodegeneration and impairment	F > M	[31,32]
	Cognitive resilience	F > M	[33]
LOAD	Rate of hippocampal volume loss	F > M	[12]
	Brain glucose hypometabolism	F > M	[34]
	Rate of cognitive decline, progression to dementia	F > M	[4,6,7,12,35,36,37,38,39]

* EOAD, early-onset AD; LOAD, late-onset AD; F, females; and M, males.

## Data Availability

Not applicable.
